# Predicting the Number of Suicides in Japan Using Internet Search Queries: Vector Autoregression Time Series Model

**DOI:** 10.2196/34016

**Published:** 2021-12-03

**Authors:** Kazuya Taira, Rikuya Hosokawa, Tomoya Itatani, Sumio Fujita

**Affiliations:** 1 Department of Human Health Sciences Graduate School of Medicine Kyoto University Kyoto Japan; 2 Division of Nursing Faculty of Health Science Institute of Medical, Pharmaceutical and Health Science Kanazawa University Kanazawa Japan; 3 Yahoo Japan Corporation Tokyo Japan

**Keywords:** suicide, internet search engine, infoveillance, query, time series analysis, vector autoregression model, COVID-19, suicide-related terms, internet, information seeking, time series, model, loneliness, mental health, prediction, Japan, behavior, trend

## Abstract

**Background:**

The number of suicides in Japan increased during the COVID-19 pandemic. Predicting the number of suicides is important to take timely preventive measures.

**Objective:**

This study aims to clarify whether the number of suicides can be predicted by suicide-related search queries used before searching for the keyword “suicide.”

**Methods:**

This study uses the infoveillance approach for suicide in Japan by search trends in search engines. The monthly number of suicides by gender, collected and published by the National Police Agency, was used as an outcome variable. The number of searches by gender with queries associated with “suicide” on “Yahoo! JAPAN Search” from January 2016 to December 2020 was used as a predictive variable. The following five phrases highly relevant to suicide were used as search terms before searching for the keyword “suicide” and extracted and used for analyses: “abuse”; “work, don’t want to go”; “company, want to quit”; “divorce”; and “no money.” The augmented Dickey-Fuller and Johansen tests were performed for the original series and to verify the existence of unit roots and cointegration for each variable, respectively. The vector autoregression model was applied to predict the number of suicides. The Breusch-Godfrey Lagrangian multiplier (BG-LM) test, autoregressive conditional heteroskedasticity Lagrangian multiplier (ARCH-LM) test, and Jarque-Bera (JB) test were used to confirm model convergence. In addition, a Granger causality test was performed for each predictive variable.

**Results:**

In the original series, unit roots were found in the trend model, whereas in the first-order difference series, both men (minimum tau 3: −9.24; max tau 3: −5.38) and women (minimum tau 3: −9.24; max tau 3: −5.38) had no unit roots for all variables. In the Johansen test, a cointegration relationship was observed among several variables. The queries used in the converged models were “divorce” for men (BG-LM test: *P*=.55; ARCH-LM test: *P*=.63; JB test: *P*=.66) and “no money” for women (BG-LM test: *P*=.17; ARCH-LM test: *P*=.15; JB test: *P*=.10). In the Granger causality test for each variable, “divorce” was significant for both men (*F*_104_=3.29; *P*=.04) and women (*F*_104_=3.23; *P*=.04).

**Conclusions:**

The number of suicides can be predicted by search queries related to the keyword “suicide.” Previous studies have reported that financial poverty and divorce are associated with suicide. The results of this study, in which search queries on “no money” and “divorce” predicted suicide, support the findings of previous studies. Further research on the economic poverty of women and those with complex problems is necessary.

## Introduction

COVID-19, which was first detected in December 2019 and was declared a pandemic by the World Health Organization in March 2020, has rapidly spread worldwide [1]. In Japan, the number of COVID-19 infections has fluctuated ever since the first person was confirmed positive in January 2020. Although the vaccination rate has been increasing, the emergence of virus variants with greater transmissibility and virulence has prolonged the pandemic [2]. The Japanese government has declared a state of emergency several times, requesting citizens to refrain from venturing out and asking restaurants and large-scale commercial facilities to close.

The limited economic activities resulting from COVID-19 restrictions have raised concerns about significant economic losses and a resultant increase in suicides [3,4]. The number of suicides in Japan, which has been decreasing since 2010, has increased rapidly since October 2020 amid the COVID-19 outbreak, especially among women [5]. Several studies have reported that employment status and economic factors are associated with suicide [6,7], which may have increased because of the impact of the pandemic on the labor market. Unlike the Lehman Brothers shock, which had a major impact on the manufacturing industry and the male labor market, the influence of COVID-19 has had a strong adverse impact on the female labor market and has been referred to as “she-cessions” [8]. Furthermore, women in Japan are likely to be at higher risk than men because they have often lagged in terms of educational standards and working conditions and have been severely affected by the pandemic [9]. In addition, increased domestic violence has been reported because of staying at home amid the COVID-19 pandemic [10].

Before the outbreak of the pandemic, the public health department of the government adopted several measures to reduce suicides. However, similar support may not be possible in the current circumstances because a large share of the human resources is earmarked for preventing COVID-19 infections. In addition, as suicide statistics can only be collated after suicide occurs and requires time for compilation, the official statistics are published only after a time lag. The cause of suicide in the official statistics is also determined based on the results of a postincident investigation by a third party such as the police; hence, official statistics cannot be used for preventive intervention. Lennon [11] demonstrated a strong correlation between unintentional injury mortality (nonsuicidal) and suicide rates, and argued that the suicide rate may be underestimated, depending on the judgment of the third party as to whether the act leading to the injury was suicidal or nonsuicidal. Therefore, preventive intervention against suicide is an important issue because it is likely that there are also potential suicides that are not captured by official statistics.

Internet search behavior has been reported to be negatively correlated with the suicide rate in the general population but positively correlated with both intentional self-harm and completed suicide in young people [12]. In Japan, internet searches for specific suicide-related terms have also been reported to be associated with the incidence of suicide among individuals aged between 20 and 30 years [13]. The negative effects of the internet on suicide generally tend to be emphasized, reflected in the term “cybersuicide” [14] originating from the phenomenon whereby suicide is encouraged when people contemplating suicide meet online. However, the internet may also help prevent suicide; for example, when suicide-related searches are performed on search engines, information on consultation desks is presented at the top of the search results, thereby helping prevent suicides [15-17].

Most previous research on suicide and queries used in internet search engines have used correlation analysis [18-22] or regression analysis [23-28]. In a correlation analysis study, Gunn and Lester [21] reported a positive correlation between suicide rates and the search terms such as “commit suicide,” “how to suicide,” and “suicide prevention,” while Sueki [20] reported a significant correlation only for “depression” and no correlation with “suicide” or “how to suicide.” Jimenez et al [19] also analyzed the correlation between 57 suicide-related words and suicide rates, and found that words such as “allergy,” “antidepressant,” “alcohol absence,” and “relationship breakdown” were significantly correlated. The studies using regression analysis also used the number of searches for words such as “suicide,” “how to commit suicide,” or “depression” to predict suicide rates. Internet search trends were reported to be associated with suicide rates for “suicide,” “depression,” and “divorce,” while Page et al [28] reported that queries such as “how to commit suicide” and “ways to kill yourself” are not straightforward indicators. In the few studies that used time series analysis, predictions were made based on direct queries such as “suicide” and “suicide methods” [29,30], and did not consider suicide-related queries or timing of searches. To prevent suicide, it is necessary to detect suicidal intent; it may be too late to do this by considering searches specifically for “suicide.” Furthermore, previous studies were published before the COVID-19 pandemic and did not generate predictions of suicide based on search queries.

A novel aspect of this study is that we construct a model to predict suicide by extracting suicide-related search words, rather than searches explicitly for the term “suicide.” Additionally, we use a vector autoregressive (VAR) model, namely, multivariate time series analysis, to examine whether the volume of search words can predict the trend toward an increasing number of suicides in Japan due to the influence of COVID-19. The results of this study will make it easier to determine the number of suicides in advance and to consider preventive measures.

## Methods

This study used the infoveillance approach for suicide in Japan by search trends in search engines.

### Measures

The monthly number of suicides collected by the National Police Agency was used as an outcome variable [5]. The data used in this study were obtained for January 2016 to March 2021 (latest available) period.

As a predictive variable, we used the number of queries associated with “suicide” from the search query log of “Yahoo! JAPAN Search,” one of the major search engines in Japan. To select queries for analysis, we first calculated the degree of association between the query “suicide” and the queries searched together with “suicide” based on the following formula for calculating a relevance score between word A and word B:



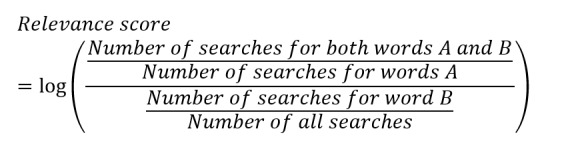



The five phrases that were used as search queries before searching for “suicide” and were highly relevant to “suicide” were extracted. These phrases were “abuse”; “work, don’t want to go”; “company, want to quit”; “divorce”; ›and “no money.” The search queries before “suicide” were used for analysis to detect trends before suicide occurrence. Monthly data from January 2016 to December 2020 were used to obtain the number of searches for the five extracted queries; this period matched that for which suicide statistics were obtained. In addition, these search numbers were tabulated by gender, and a correction was applied to adjust for the sex ratio in the Japanese population, as follows:



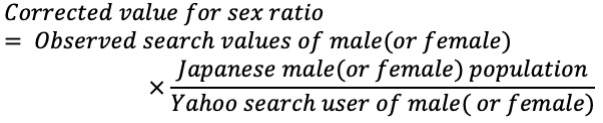



### Statistical Analysis

The augmented Dickey-Fuller (ADF) test, a unit root test, was performed to verify the stationarity of each variable used in the analysis. ADF tests were conducted in the trend model, which assumed a time trend term and constant term, and the drift model, which assumed only a constant term. As the time series data with unit roots becomes steady in many cases by taking a difference, the ADF test is performed on the difference variable. The lag order was selected by checking the convergence of the model while making decisions based on Akaike information criterion.

Johansen test was performed to verify the existence of cointegration (a relationship in which the linear sum of two unit-root processes becomes a stationary process) between each variable. The variables in this study were confirmed to have cointegration, and all series of the first-order differences resulted in stationary processes; therefore, we used VAR models with first-difference processes. The VAR model is a multivariate time series analysis, developed based on the philosophy of “let the data speak for themselves (i.e. measurement without theory).” It has high prediction accuracy and has been widely recognized in the field of macroeconomic models [31]. The VAR model is most suitable for this study as a method for multivariate time series analysis with high prediction accuracy using search data of search engines selected without theoretical background. Confirming the VAR model convergence necessitates confirming whether it satisfies the following three standard assumptions for the disturbance term (residual): (1) does not have serial correlation (autocorrelation), (2) has a uniform dispersion, and (3) has a normal distribution. We performed the Breusch-Godfrey Lagrangian multiplier (BG-LM) test (null hypothesis [H0]: no serial correlation), the autoregressive conditional heteroskedasticity Lagrangian multiplier (ARCH-LM) test (H0: uniform dispersion), and the Jarque-Bera (JB) test (H0: normal distribution) to confirm serial noncorrelation, uniform dispersion, and normal distribution of the disturbance term, respectively. All analyses in this study were performed using R version 3.6.2 (R Foundation for Statistical Computing). This study involved secondary analysis of public statistics and anonymized existing data; therefore, ethical approval by an ethics committee was not required.

## Results

### Confirmation of Unit Root and Cointegration Relationship

According to the ADF test, the null hypothesis of unit root existence for the variables “suicide” and “company, want to quit” in men and “suicide”; “divorce”; “no money”; and “company, want to quit” in women could not be rejected in the original series. In the first-order difference series, the null hypothesis was rejected for all variables for both men and women (Tables 1 and 2). In the Johansen test, “divorce” and “company, want to quit” were adopted for both men and women as the null hypothesis of r=0 (no cointegration) based on 10% of the critical values, but other variables were rejected. When r=1 (cointegration rank 1), the null hypothesis was adopted for all the variables (Table 3).

**Table 1:** Results of the augmented Dickey-Fuller test (original series).

			Male										Female							
			Trend				Drift					Trend						Drift		
			Lag		Tau3		Lag		Tau2		Lag				Tau 3		Lag			Tau 2
The number of suicides			1		−3.29^a^		1		−2.74^b^		1				−2.34		1			−2.38
**Search number of**																				
	“Abuse”	1		−5.80^c^		1		−5.86^d^		1				−5.98^c^		1			−5.94^d^	
	“Divorce”	1		−2.34		1		−2.62^b^		1				−2.96		1			−3.02^e^	
	“No money”	1		−4.29^c^		1		−3.16^e^		1				−3.34^a^		1			−2.32	
	“Work, don’t want to go”	1		−3.92^f^		1		−3.82^d^		1				−3.65^c^		1			−3.25^e^	
	“Company, want to quit”	1		−4.53^c^		1		−2.35		1				−4.46^c^		1			−1.85	

^a^Trend model critical value 10%=–3.15.

^b^Drift model critical value 10%=–2.58.

^c^Trend model critical value 1%=–4.04.

^d^Drift model critical value 1%=–3.51.

^e^Drift model critical value 5%=–2.89.

^f^Trend model critical value 5%=–3.45.

**Table 2:** Results of the augmented Dickey-Fuller test (first-order difference series).

			Male								Female						
			Trend				Drift				Trend				Drift		
			Lag		Tau3		Lag		Tau2		Lag		Tau 3		Lag		Tau 2
The number of suicides			1		–5.91^a^		1		–5.94^b^		1		–3.81^c^		1		–3.90^b^
**Search number of**																	
	“Abuse”	1		–9.24^a^		1		–9.33^b^		1		–9.42^a^		1		–9.50^b^	
	“Divorce”	1		–5.38^a^		1		–5.28^b^		1		–5.87^a^		1		–5.82^b^	
	“No money”	1		­–7.73^a^		1		–7.80^b^		1		–6.72^a^		1		–6.77^b^	
	“Work, don’t want to go”	1		–7.64^a^		1		–7.60^b^		1		–7.01^a^		1		–6.96^b^	
	“Company, want to quit”	1		–7.12^a^		1		–7.19^b^		1		–7.71^a^		1		–7.71^b^	

^a^Trend model critical value 1%=–4.04.

^b^Drift model critical value 1%=–3.51.

^c^Trend model critical value 5%=–3.45.

**Table 3:** Results of Johansen (cointegration) tests, including the trend term and a seasonal dummy variable, between the number of suicides and each search query.

Variables and H_0_				Lags		Test statistics		Critical values				
								10%		5%		1%
**Male**												
	**Search number of “Abuse”**			2								
		r≤1			10.82^a^		10.49		12.25		16.26	
		r=0			41.21^b^		22.76		25.32		30.45	
	**Search number of “Divorce”**			3								
		r≤1			4.65		10.49		12.25		16.26	
		r=0			26.01^c^		22.76		25.32		30.45	
	**Search number of “No money”**			5								
		r≤1			5.99		10.49		12.25		16.26	
		r=0			34.66^b^		22.76		25.32		30.45	
	**Search number of “Work, don’t want to go”**			5								
		r≤1			12.52^c^		10.49		12.25		16.26	
		r=0			35.49^b^		22.76		25.32		30.45	
	**Search number of “Company, want to quit”**			2								
		r≤1			12.08^a^		10.49		12.25		16.26	
		r=0			29.45^c^		22.76		25.32		30.45	
**Female**												
	**Search number of “Abuse”**			3								
		r≤1			15.63^c^		10.49		12.25		16.26	
		r=0			36.50^b^		22.76		25.32		30.45	
	**Search number of “Divorce”**			3								
		r≤1			4.23		10.49		12.25		16.26	
		r=0			25.09^c^		22.76		25.32		30.45	
	**Search number of “No money”**			5								
		r≤1			3.07		10.49		12.25		16.26	
		r=0			32.00^b^		22.76		25.32		30.45	
	**Search number of “Work, don’t want to go”**			5								
		r≤1			9.72		10.49		12.25		16.26	
		r=0			35.27^b^		22.76		25.32		30.45	
	**Search number of “Company, want to quit”**			3								
		r≤1			12.99^c^		10.49		12.25		16.26	
		r=0			30.35^c^		22.76		25.32		30.45	

^a^>1%.

^b^>10%

^c^>5%.

### Prediction of the Number of Suicides by the VAR Model and Granger Causality Test

**Figure 1:**
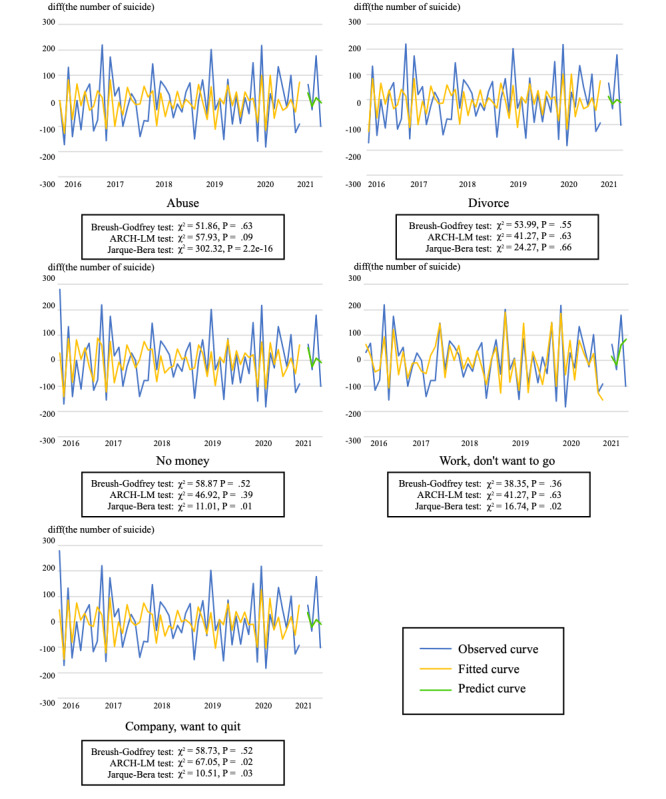
Changes in the number of suicides and predicted values of vector autoregression models using each search query (men). ARCH-LM: autoregressive conditional heteroscedasticity Lagrangian multiplier.

**Figure 2:**
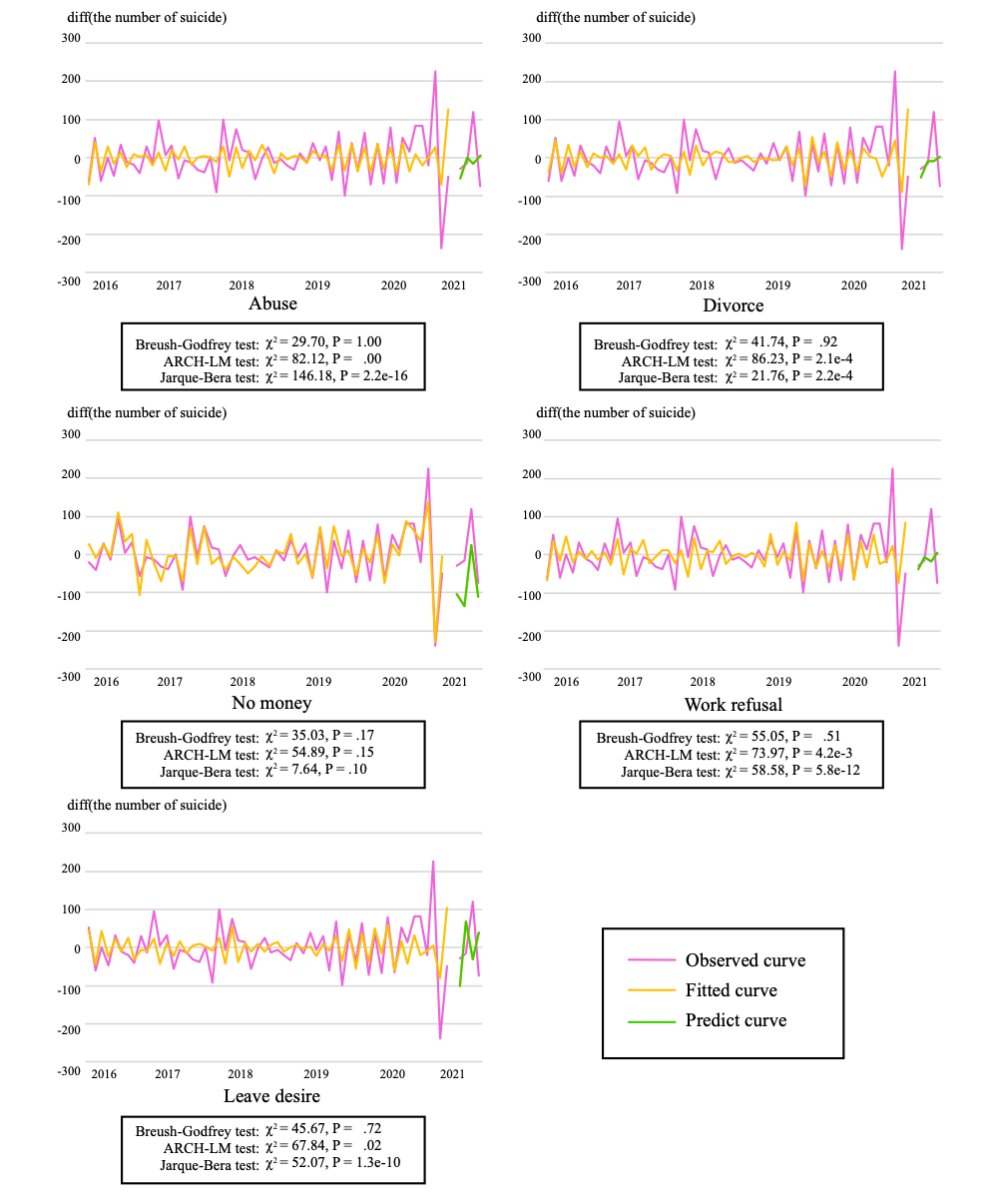
Changes in the number of suicides and predicted values of vector autoregressive models using each search query (women). ARCH-LM: autoregressive conditional heteroscedasticity Lagrangian multiplier.

From the aforementioned results, as all first-order difference series were standing waves and there were variables with a first-order cointegration relationship, a VAR model using the first-order difference series was designed for each gender. Figures 1 and 2 plot a VAR model constructed using data from January 2016 to December 2020 and the number of suicides from January 2021 to March 2021 predicted by the model for men and women, respectively.

For the BG-LM, ARCH-LM, and JB tests performed to confirm whether the model converged, the variables that converged at the 5% level were “divorce” for men (BG-LM: χ^2^_20_=53.99, *P*=.55; ARCH-LM: χ^2^_45_=41.27, *P*=.63; JB: χ^2^_2_=24.27, *P*=.66) and “no money” for women (BG-LM: χ^2^_20_=35.03, *P*=.17; ARCH-LM: χ^2^_45_=54.89, *P*=.15; JB: χ^2^_2_=7.64, *P*=.10). Furthermore, at the 1% level, the model for men converged for “no money” (BG-LM: χ^2^_20_=58.87, *P*=.52; ARCH-LM: χ^2^_45_=46.92, *P*=.39; JB: χ^2^_2_=11.01, *P*=.01); “work, don’t want to go” (BG-LM: χ^2^_20_=38.35, *P*=.36; ARCH-LM: χ^2^_45_=41.27, *P*=.63; JB: χ^2^_2_=16.74, *P*=.02); and “company, want to quit” (BG-LM: χ^2^_20_=58.73, *P*=.52; ARCH-LM: χ^2^_45_=67.05, *P*=.02; JB: χ^2^_2_=10.51, *P*=.03), but only “no money” converged for women—a result that is the same as that at the 5% level. In the Granger causality test for each variable (Table 4), “divorce” was significant at the 5% level for both men (*F*_104_=3.29; *P*=.04) and women (*F*_104_=3.23; *P*=.04).

**Table 4:** Result of Granger causality test of each search query for the number of suicides.

		Male			Female	
		*F* test (*df*)	*P* value	*F* test (*df*)		*P* value
**Search number of**						
	“Abuse”	0.238 (102)	.79	0.237 (104)		.76
	“Divorce”	3.290 (104)	.04^a^	3.229 (104)		.04^a^
	“No money”	0.752 (110)	.39	0.736 (62)		.68
	“Work, don’t want to go”	0.840 (74)	.56	1.641 (104)		.20
	“Company, want to quit”	3.760 (110)	.06	1.028 (98)		.38

^a^*P*<.05.

## Discussion

### Principal Results

The models using the number of searches for the term “divorce” for men and “no money” for women converged best among the search queries used in this study to predict the number of suicides. In Figures 1 and 2, in the convergent model, both the VAR model and the predicted value using the model fit well the measured value. This result indicates that the model, based not only on the search query “suicide” but also on the queries related to “suicide,” was effective at predicting the number of suicides.

Further, the model with the query “no money” converged best for women, with an increasing number of suicides during the COVID-19 pandemic. In recent years, “invisible” poverty has been reported to have become more severe among the younger generation, especially among single-mother households in Japan [32-34]. Furthermore, compared to the economic downturn caused by the Lehman Brothers shock, which had a large impact on males in the manufacturing industry, the economic downturn caused by the COVID-19 pandemic has had a large impact on females and is sometimes referred to as she-cession [8,35]. A decrease of 700,000 female workers against 390,000 male workers has occurred in Japan since the COVID-19 pandemic began. The reason is that more than half of the female employees are nonregular employees who are engaged in industries that have been severely impacted by the pandemic—food service, life-related service, entertainment, and retail industries [35,36]. The increase in suicide among women in Japan may be attributed to the potential economic problems of disadvantaged women. For the same reason, although the variation in suicide projections was smaller for men than for women, given the good convergence test results, the influence of COVID-19 is smaller on men than on women, and a future gradual increase in suicides may be observed among men.

By contrast, “Analysis of Suicides in Coronavirus (Emergency Report)” [37] published by the Japan Suicide Countermeasures Promotion Center indicated that the number of suicides among “women with housemates” and “unemployed women” increased substantially. In addition, the report also suggests that various problems such as domestic violence, childcare concerns, mental illness, nursing care fatigue, and the Werther effect—an increase in the number of suicides because of reports of famous people committing suicide—as contributing factors. Regarding the query “no money” (which was a good predictor of women’s suicide in this study), namely, economic poverty, the background of the poverty and the problems associated with poverty were not considered, which is a topic that requires further research.

Regarding future work, it is desirable to conduct a study of the effectiveness of long-term forecasts and to consider economic indicators other than those related to search queries (eg, searches for “poverty” and “unemployment”). This would enable a practical prediction model to be developed that would be useful for policy decision-making.

### Limitations

This study has several limitations. First, the age at which people commit suicide versus the age at which they search for suicide-related information may differ. However, the number of searches used in this study included searches using personal computers, tablets, and smartphones. Considering that the smartphone and personal computer penetration rates in Japan in 2020 were 86.8% and 68.1%, respectively [38], most of the searches by each age group can be considered to have been covered. Second, the Metropolitan Police Department’s suicide statistics used in the study include provisional figures and are compiled based on the address of the place where the person committed suicide, not the place where the person lived. Bias might therefore exist, as the number of suicides is relatively high in areas where mass suicides occur or in locations famous for suicides. Amid the COVID-19 pandemic, the impact of economic shocks on suicide may be moderate because the government has been providing financial support and enforcing behavioral restrictions on its citizens. The economic impact could be even stronger if government support changes or if COVID-19’s impact persists in the future. The predictions in this study do not consider government support during the pandemic and may overstate the actual number.

### Comparison With Prior Work

The results of this study support the results of previous studies related to suicide but are novel in that they were demonstrated using search behavior on the internet. For men, search queries such as “no money”; “work, don’t want to go”; and “company, want to quit” were also significant at the 1% level, consistent with previous studies in which economic indicators and employment status were associated with suicide [6,7]. In Japan, the employment rate of men is higher than that of women culturally, and the suicide rate is also higher for men. Since the early 1980s, the word “Karoshi,” which means a permanent inability to work or death because of acute ischemic heart disease caused by excessive work overload and suicides because of mental disorders caused by overwork, has been created and reported in Japan [39,40]. Therefore, the fact that the search queries related to employment were associated with men may represent a characteristic of Japan.

In the Granger causality test, the query “divorce” was significant for both genders. In Western countries such as the United States and Canada, divorce has been reported to be a risk factor for suicide, particularly among men [41,42]. However, the same tendency reportedly cannot be replicated in Japan. Although the prediction model for the number of suicides did not converge well, divorce may also be an important factor associated with suicide in Japan [43] and requires further investigation.

### Conclusions

In this study, we found that the trend in the number of suicides could be predicted using search queries related to suicide that occurred before searching for the keyword “suicide.” The queries that converged in the prediction model for the number of suicides were “divorce” for men and “no money” for women. As of September 2021, the pandemic situation in Japan and the world persists because of the emergence of variants of concern and adverse economic effects, and an increase in the number of suicides is predicted. Further research on the situation of women living in economic poverty and having complex problems and considering mechanisms to support them amid the COVID-19 pandemic—which has severely impacted them—is necessary.

## Abbreviations

ADF: augmented Dickey-Fuller
ARCH-LM: autoregressive conditional heteroskedasticity Lagrangian multiplier
BG-LM: Breusch-Godfrey Lagrangian multiplier
JB: Jarque-Bera
VAR: vector autoregressive
